# Knowledge, attitudes, and practices of patients with endometriosis regarding endometriosis surgery and postoperative care in Liaoning Province, China: a cross-sectional analysis

**DOI:** 10.1186/s12884-025-07852-1

**Published:** 2025-07-28

**Authors:** Lei Xu, Nan Jiang, Xiaoxin Xiu

**Affiliations:** Department of Gynecologic, the First Affiliated Hospital of Dalian Medical University, Dalian, 116011 China

**Keywords:** Endometriosis, Postoperative care, Knowledge, Attitude, Practice

## Abstract

**Background:**

Surgical intervention is the main treatment for endometriosis, and effective postoperative care is crucial for long-term health. This study examined the knowledge, attitudes, and practices (KAP) of patients with endometriosis in Liaoning Province, China, regarding surgery and postoperative care.

**Methods:**

This cross-sectional study included patients with endometriosis at the First Affiliated Hospital of Dalian Medical University from July 2024 to February 2025. Participants provided informed consent, and a self-designed questionnaire was used collected socio-demographic data and assessed KAP scores.

**Results:**

A total of 417 endometriosis patients completed the survey, with a mean age of 35.34 ± 9.22 years and a response rate of 99.04%. The mean scores for knowledge, attitudes, and practices were 13.73 ± 3.97 (possible range: 0–30), 41.65 ± 3.31 (possible range: 11–55), and 28.22 ± 3.18 (possible range: 7–35), respectively. Positive correlations were observed between knowledge and attitude (*r* = 0.105, *P* = 0.033), knowledge and practice (*r* = 0.175, *P* < 0.001), and attitude and practice (*r* = 0.100, *P* = 0.041). Multivariate Logistic analysis revealed that knowledge (OR = 1.11, 95% CI: 1.05–1.17, *P* < 0.001) was positively associated with practice, while age showed a negative relationship (OR = 0.97, 95% CI: 0.95–0.99, *P* = 0.008). SEM analysis indicated a direct effect of knowledge on attitude (β = 0.587, *P* = 0.003).

**Conclusions:**

Patients with endometriosis in Liaoning showed significant knowledge gaps but had positive attitudes and practice towards surgery and postoperative care. Educational interventions are essential to improve their understanding and reinforce these attitudes.

**Supplementary Information:**

The online version contains supplementary material available at 10.1186/s12884-025-07852-1.

## Background

Endometriosis is a chronic, estrogen-dependent gynecological disorder characterized by the presence of endometrial-like tissue outside the uterine cavity [1]. It affects 5–10% of women of reproductive age (typically defined as 15–49 years) globally, which corresponds to over 176 million individuals [2]. Notably, there are significant regional disparities in incidence; for instance, in 2019, the age-standardized incidence rate (ASIR) was 30 per 100,000 in Africa and 35 per 100,000 in South America [3]. It is important to note that ASIR reflects incidence rather than prevalence, and these values indicate newly diagnosed cases rather than the total disease burden. This distinction is critical, as incidence data can highlight diagnostic capacity and healthcare access disparities between regions. In China, an estimated 4.19% of women aged 15–49 years are affected, based on a cross-sectional national study [4]; given differences in diagnostic methods and population sampling, the global and Chinese prevalence figures may not be directly comparable. This lower prevalence compared to global figures may be attributed to underdiagnosis, which arises from limited access to laparoscopic confirmation and cultural stigma surrounding the reporting of gynecological symptoms [5, 6]. Furthermore, the lack of large-scale epidemiological studies in China likely contributes to an underestimation of the disease burden [7].

Endometriosis is characterized by adhesions, fibrosis, and organ dysfunction, leading to chronic pain and complications affecting bowel and urinary function [8, 9]. In addition to physical morbidity, patients experience heightened risks of mental health disorders, particularly depression and anxiety [10]. While hormonal therapies and analgesics offer some relief, surgical intervention remains the most effective treatment option [11]. The success of surgical outcomes is contingent not only upon surgical precision but also on comprehensive postoperative management and long-term follow-up [12]. The effectiveness of surgery varies significantly, with many patients experiencing pain recurrence and necessitating reoperation [13]. The postoperative recurrence rate of endometriosis—defined as the return of symptoms or lesions after surgical treatment—can reach 50% [14], particularly in patients with deep infiltrating endometriosis (DIE) [15]. Without a standardized medication regimen, the recurrence rate in the first year is 15–30%, highlighting the importance of long-term management [14]. Adherence is affected by patient knowledge, socioeconomic status, and cultural beliefs [16, 17], with rural patients often lacking understanding—only 12.3% show adequate awareness. Cultural beliefs that prioritize enduring pain also may be associated with a higher risk of recurrence [14]. Targeted health education can significantly enhance compliance, evidenced by a 50% increase in adherence to postoperative activity restrictions after preoperative education [15].

The KAP model—a behavioral framework suggesting that knowledge influences attitudes, which in turn shape practices—has been widely used to understand patient behavior in chronic disease contexts such as diabetes, hypertension, and HIV. While surgical technique and disease severity are critical factors, emerging evidence emphasizes the importance of patients’ knowledge, attitudes, and practices (KAP) regarding surgery and recovery. A thorough understanding of procedures, outcomes, and complications enables patients to align their expectations with reality and adhere to postoperative guidelines [18]. Supportive attitudes toward recovery directly influence compliance with activity restrictions, pain management, and follow-up attendance, all of which are essential for minimizing recurrence [19]. In Liaoning Province, China, disparities in healthcare access significantly complicate postoperative outcomes [20]. Numerous patients encounter barriers, including delayed diagnoses and restricted access to specialists, while entrenched cultural beliefs may impede symptom disclosure and the timely pursuit of treatment [21]. These systemic and sociocultural factors likely interact with individual KAP, thereby perpetuating a cycle of inadequate care. For example, a lack of understanding regarding endometriosis within rural communities can reinforce negative attitudes toward surgical interventions, consequently diminishing adherence to postoperative care practices [22]. Understanding these dynamics is may be important for designing targeted interventions aimed at improving reproductive health equity. The KAP model, a well-established public health framework, posits that knowledge shapes attitudes, which in turn drive behaviors [23]. However, existing KAP research on endometriosis has disproportionately focused on healthcare providers rather than patients. For instance, a study in Denmark found that gynecological nurses often stereotype endometriosis patients based on subjective assumptions [24]. In contrast, patient-centered KAP evidence—particularly concerning surgical experiences, long-term pain management, and behavioral compliance—remains limited. This gap is significant, as patients’ knowledge, attitudes, and practices fundamentally influence postoperative success.

This study aimed to examine the deficiencies in KAP regarding surgical procedures and postoperative care among endometriosis patients in Liaoning Province. It was hypothesized that, in accordance with KAP theory, an enhancement in patient knowledge will correlate with a more favorable attitude toward surgical interventions, thereby contributing to improved adherence to postoperative practices. Given the resource-constrained context of Liaoning, the implementation of KAP-informed patient education strategies may serve to effectively bridge existing knowledge gaps, optimize postoperative management, and ultimately improve long-term health outcomes.

## Methods

### Study design and participants

This cross-sectional study was conducted among endometriosis patients at the First Affiliated Hospital of Dalian Medical University between July 2024 and February 2025. Ethical approval was granted by the Medical Ethics Committee of the First Affiliated Hospital of Dalian Medical University (Approval No: PJ-KS-KY-2024-580), and informed consent was obtained from all participants prior to the administration of the survey. Inclusion criteria were: (1) female patients aged 18 or older; (2) a confirmed diagnosis of endometriosis. Exclusion criteria included: (1) not agreeing to participate; (2) having cognitive impairment; (3) having impaired consciousness or psychiatric disorders.

### Procedures

**Fig. 1:**
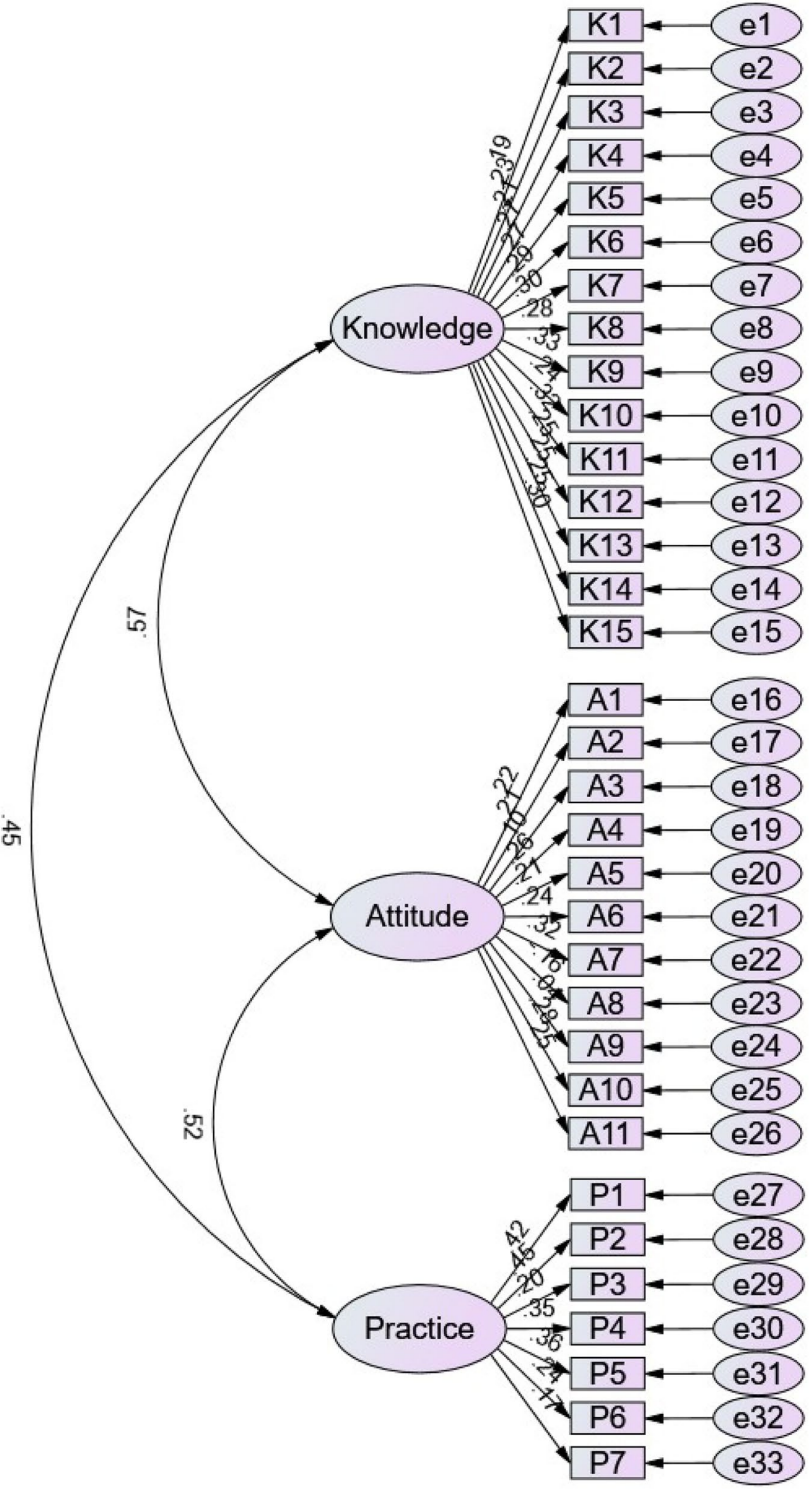
Confirmatory factor analysis results of KAP questionnaire

The questionnaire utilized in this study was meticulously designed based on the “Chinese Consensus on Long-Term Management of Endometriosis” and the “Expert Recommendations on Long-Term Management of Endometriosis Following Laparoscopic Surgery” and “Management of Endometriosis Surgery Complications” [25–27]. A pilot study involving 34 patients diagnosed with endometriosis at the First Affiliated Hospital of Dalian Medical University demonstrated satisfactory internal consistency, as evidenced by an overall Cronbach’s alpha coefficient of 0.928. Specifically, the knowledge dimension exhibited a coefficient of 0.941, the attitude dimension 0.847, and the practice dimension 0.842. The survey instrument underwent three iterative rounds of modification, integrating feedback from three subject matter experts to enhance the quantity, complexity, and breadth of the questions. The final questionnaire, developed in Chinese, consisted of four dimensions: The demographic information consisted of 13 items (including variables such as age, height, weight, educational attainment, income, marital status, smoking habits, alcohol consumption, and prior medical history). The remaining sections evaluated knowledge, attitudes, and practices. The knowledge dimension comprised 15 items, each evaluated on a scale where participants received 2 points for “Very familiar,” 1 point for “Heard of,” and 0 points for “Unclear,” resulting in a potential total score ranging from 0 to 30 points. The attitude dimension comprised 11 items assessed using a five-point Likert scale, with response options ranging from “Strongly agree” to “Strongly disagree,” corresponding to scores from 5 to 1. Notably, for items 8 and 9, the scoring system was inverted, assigning scores from 1 to 5. This reverse scoring was applied because both items were negatively worded, and inversion ensured consistency in interpreting higher scores as more positive attitudes. Consequently, the total possible score for this dimension ranged from 11 to 55 points. The practice dimension consisted of 7 questions, scored from 5 to 1 based on responses ranging from “Always” to “Never,” allowing for a score range of 7 to 35 points. These items covered behaviors such as medication adherence, attending follow-ups, and participation in educational activities. Scores exceeding 75% of the maximum possible in each dimension indicated sufficient knowledge, a positive attitude, and proactive practices [28]. The CFA demonstrated strong model fit for the hypothesized structure (CMIN/DF value: 1.064, RMSEA value: 0.012, IFI value: 0.911, TLI value: 0.894, and CFI value: 0.901), validating the structural coherence of the measurement model and its alignment with the theoretical framework (Supplementary Table 1, Table 2; Fig. 1).

**Table 1:** Demographic characteristics and knowledge, attitude, and practice scores

Variables	*N* (%)	Knowledge, mean ± SD	*P*	Attitude, mean ± SD	*P*	Practice, mean ± SD	*P*
Total	413	13.73 ± 3.97		41.65 ± 3.31		28.22 ± 3.18	
Age (years)	35.34 ± 9.22						
Education level			0.441		0.012		0.791
Senior high school/technical secondary school or below	124 (30.02)	13.27 ± 3.23		40.90 ± 3.51		27.98 ± 3.61	
Associate/Bachelor’s degree or above	289 (69.98)	13.92 ± 4.23		41.98 ± 3.17		28.33 ± 2.97	
Ethnicity			0.912		0.712		0.681
Han	392 (94.92)	13.72 ± 3.97		41.67 ± 3.29		28.25 ± 3.15	
Minority	21 (5.08)	13.86 ± 4.11		41.38 ± 3.71		27.71 ± 3.69	
Employment status			0.840		0.175		0.995
Employed	289 (69.98)	13.78 ± 4.13		41.83 ± 3.25		28.22 ± 3.29	
Other	124 (30.02)	13.60 ± 3.56		41.24 ± 3.42		28.21 ± 2.90	
Monthly income (Chinese Yuan)			0.222		0.732		0.053
Equivalent to <$280 USD	25 (6.05)	12.72 ± 3.73		41.92 ± 4.01		28.64 ± 3.88	
Equivalent to ~$280–700 USD	88 (21.31)	13.50 ± 3.66		41.88 ± 2.79		27.75 ± 3.16	
Equivalent to ~$700–1,400 USD	186 (45.04)	13.50 ± 3.67		41.69 ± 3.39		28.75 ± 2.84	
Equivalent to ~$1,400–2,800 USD	41 (9.93)	13.93 ± 4.21		40.93 ± 3.39		27.34 ± 3.38	
Equivalent to > 2,800 USD	60 (14.53)	14.83 ± 3.49		41.57 ± 3.07		28.13 ± 2.83	
Prefer not to disclose	13 (3.15)	14.69 ± 8.88		41.69 ± 4.71		26.23 ± 5.42	
Marital status			0.432		0.602		0.351
Single	143 (34.62)	13.99 ± 3.86		41.79 ± 3.23		28.52 ± 2.99	
Married	270 (65.38)	13.59 ± 4.02		41.58 ± 3.35		28.06 ± 3.26	
Having children			0.642		0.752		0.247
Yes	194 (46.97)	13.75 ± 4.08		41.52 ± 3.26		27.91 ± 3.44	
No	219 (53.03)	13.70 ± 3.87		41.77 ± 3.35		28.49 ± 2.90	
Smoking			0.009		0.111		0.458
No	379 (91.99)	13.84 ± 4.02		41.72 ± 3.32		28.24 ± 3.25	
Yes	33 (7.99)	12.39 ± 3.08		40.85 ± 3.04		28.00 ± 2.19	
Alcohol consumption			0.548		0.563		0.662
No	250 (60.68)	13.82 ± 4.06		41.57 ± 3.29		28.12 ± 3.32	
Yes	162(39.23)	13.58 ± 3.83		41.78 ± 3.35		28.37 ± 2.95	
Medical insurance			0.428		0.463		0.603
Yes	392 (94.92)	13.81 ± 3.87		41.69 ± 3.29		28.24 ± 3.15	
No	21 (5.08)	12.19 ± 5.44		40.86 ± 3.58		27.86 ± 3.62	
Duration of endometriosis			0.416		0.721		0.660
< 3 years	298 (72.15)	13.57 ± 4.08		41.65 ± 3.33		28.28 ± 3.17	
> 3 years	115 (27.85)	14.14 ± 3.64		41.65 ± 3.27		28.07 ± 3.21	
Female relatives with endometriosis			0.715		0.212		0.276
Yes	61 (14.77)	13.57 ± 2.73		42.20 ± 3.36		28.66 ± 2.64	
No	281 (68.28)	13.82 ± 4.28		41.50 ± 3.30		28.02 ± 3.39	
Not sure	70 (16.95)	13.50 ± 3.59		41.80 ± 3.27		28.63 ± 2.64	
Surgical treatment for endometriosis			0.058		0.117		0.549
Yes	292 (70.70)	14.04 ± 4.04		41.83 ± 3.35		28.16 ± 3.22	
No	121 (29.30)	12.96 ± 3.68		41.23 ± 3.19		28.36 ± 3.09	

### Questionnaire distribution and quality control

The questionnaire was primarily disseminated through online channels, specifically targeting patients diagnosed with endometriosis at the hospital. The Sojump platform (https://www.wjx.cn/) was utilized to generate a QR code for the electronic surveys. The research team delineated the objectives of the study to the Heads of five pivotal clinical departments involved in the management of endometriosis patients: Gynecology, Reproductive Medicine, General Surgery, Pain Management, and Urology. This communication facilitated their consent and collaboration. The Heads of Departments (HODs) assumed a critical dual role in the support of the study: first, they provided administrative approval for patient recruitment and data collection within their respective departments; second, they contributed to the establishment of patient trust and enhanced compliance with the questionnaires by personally endorsing the research during standardized health education sessions. A convenience sampling approach was employed to select endometriosis patients from the First Affiliated Hospital of Dalian Medical University. Between July 2024 and February 2025, the research team engaged in direct interaction with all admitted endometriosis patients. Participants received a comprehensive explanation of the study’s aims and methodologies. A QR code was provided to facilitate access to the electronic questionnaire. To mitigate the risk of duplicate responses, an IP address restriction was implemented, ensuring that each survey could be completed only once per IP address. Following data collection, the research team conducted a quality assessment of the completed questionnaires. Responses that were submitted in less than 120 s, as well as those exhibiting clear logical inconsistencies or patterns of repetitive answers, were deemed invalid and subsequently excluded from the analysis. The 120-second threshold was applied to help identify inattentive or insincere responses.

### Sample size

Sample size was calculated using the formula for a cross-sectional study [29]: $\:\alpha\:$ = 0.05, $\:\text{n}={\left(\frac{{Z}_{1-\alpha\:/2}}{\delta\:}\right)}^{2}\times\:p\times\:\left(1-p\right)$ where $\:{Z}_{1-\alpha\:/2}$=1.96 when $\:\alpha\:$ = 0.05, the assumed degree of variability of $\:p$ = 0.5 maximises the required sample size, and δ represents the margin of error (precision), which was determined as 0.05, and at least 330 participants should be required. To accommodate an anticipated response rate of 80% (1–0.20), the initial sample size of 330 was adjusted, yielding an updated requirement of 330/0.80 = 412.5, which was subsequently rounded up to 413 participants.

### Statistical analysis

Statistical analyses were performed utilizing the Statistical Package for the Social Sciences (SPSS) version 27.0 and Analysis of Moment Structures (AMOS) version 26.0, both developed by IBM Corp. in Armonk, NY, USA. Continuous variables were assessed for normality and reported as means with standard deviations (SD) or medians with interquartile ranges (IQR), contingent upon their distribution. Utilize the Bootstrap method to conduct 1,000 repetitions of sampling for all datasets, and subsequently calculate the 95% confidence interval (CI) for the mean of KAP. Categorical variables were presented as frequencies and percentages (n, %). For continuous variables exhibiting a normal distribution, comparisons were conducted using independent sample t-tests or analysis of variance (ANOVA). In contrast, variables characterized by skewed distributions were analyzed using the Wilcoxon-Mann-Whitney test or Kruskal-Wallis ANOVA. Correlation analyses employed either Pearson or Spearman correlation coefficients, depending on the distribution of the data. Variables with a P-value of less than 0.05 in univariate analyses were subsequently included in the multivariate regression analyses. Structural equation modeling (SEM) was utilized to investigate the interrelationships among the constructs of knowledge (K), attitudes (A), and practices (P). The hypotheses posited by the SEM framework were as follows: (1) knowledge directly influences attitude, (2) attitude directly influences practice, and (3) knowledge influences practice both directly and indirectly. These pathways are theoretically grounded in the KAP model, which suggests that knowledge shapes attitudes and subsequently influences behaviors [23]. An acceptable model fit was established in accordance with established criteria. Furthermore, confirmatory factor analysis (CFA) was performed to validate the measurement structure of the questionnaire and to assess the construct validity of the KAP model. The evaluation of the factor structure encompassed standardized factor loadings, critical ratios (C.R.), and fit indices, including the Root Mean Square Error of Approximation (RMSEA), Comparative Fit Index (CFI), Tucker-Lewis Index (TLI), and Incremental Fit Index (IFI). All statistical tests were two-sided, and P-values < 0.05 were considered statistically significant.

## Results

### Demographic characteristics

A total of 417 participants completed the survey; however, four responses were excluded from analysis due to completion times of less than 120 s, resulting in a valid response rate of 99.04%. The mean age of participants was 35.34 ± 9.22 years. The majority of participants held an associate’s or bachelor’s degree or higher (69.98%), identified as Han ethnicity (94.92%), were employed (69.98%), and did not have children (53.03%). Additionally, 45.04% reported a monthly income ranging from 5,000 to 10,000 RMB Yuan (approximately USD 700–1,400 based on the 2024 exchange rate). A significant majority of participants did not smoke (91.99%) or consume alcohol (60.68%). Furthermore, a notable percentage (72.15%) had been diagnosed with endometriosis for less than three years, and 70.70% had undergone surgical treatment for the condition (Table 1).

**Table 2:** Univariate and multivariate logistic regression analysis of practice

	Univariate logistic analysis		Multivariate logistic analysis	
	OR (95% CI)	*P*	OR (95% CI)	*P*
Knowledge score	1.12 (1.06, 1.18)	< 0.001	1.11 (1.05, 1.17)	< 0.001
Attitude score	1.07 (1.01, 1.14)	0.033	1.05 (0.98, 1.12)	0.152
Age (years)	0.97 (0.95, 0.99)	0.007	0.97 (0.95, 0.99)	0.008
Education level				
Senior high school/technical secondary school or below	Ref.			
Associate/Bachelor’s degree or above	1.22 (0.80, 1.88)	0.357		
Ethnicity				
Han	Ref.			
Minority	0.99 (0.40, 2.43)	0.975		
Employment status				
Employed	Ref.			
Other	1.15 (0.74, 1.78)	0.530		
Monthly income (RMB Yuan)				
Equivalent to <$280 USD	Ref.			
Equivalent to ~$280–700 USD	0.96 (0.39, 2.38)	0.935		
Equivalent to ~$700–1,400 USD	1.33 (0.57, 3.14)	0.510		
Equivalent to ~$1,400–2,800 USD	0.85 (0.31, 2.34)	0.756		
Equivalent to > 2,800 USD	1.07 (0.41, 2.79)	0.886		
Prefer not to disclose	0.57 (0.15, 2.21)	0.417		
Marital status				
Single	Ref.			
Married	0.72 (0.47, 1.11)	0.135		
Having children				
Yes	Ref.			
No	1.27 (0.85, 1.89)	0.245		
Smoking				
No	Ref.			
Yes	0.93 (0.45, 1.92)	0.841		
Alcohol consumption				
No	Ref.			
Yes	0.97 (0.64, 1.45)	0.866		
Medical insurance				
Yes	Ref.			
No	0.65 (0.27, 1.57)	0.343		
Duration of endometriosis				
< 3 years	Ref.			
> 3 years	0.92 (0.59, 1.44)	0.724		
Female relatives with endometriosis				
Yes	Ref.			
No	0.75 (0.42, 1.35)	0.339		
Not sure	0.88 (0.43, 1.81)	0.725		
Surgical treatment for endometriosis				
Yes	Ref.			
No	1.20 (0.77, 1.87)	0.409		

### Knowledge, attitude, and practice dimensions

The mean scores for knowledge, attitudes, and practices were reported as follows: knowledge (13.73 ± 3.97, 95% CI [13.35, 14.12], range: 0–30), attitudes (41.65 ± 3.31, 95% CI [41.36, 41.98], range: 11–55), and practices (28.22 ± 3.18, 95% CI [27.91, 28.55], range: 7–35). Familiarity with knowledge items varied significantly, ranging from 3.63 to 53.27%. The lowest level of familiarity (3.63%) was associated with the statement that the primary symptom of endometriosis is progressively worsening secondary dysmenorrhea, which predominantly affects women aged 25 to 45 years (K2). Furthermore, only 12.11% of participants accurately recognized that endometriosis is a chronic condition with an infertility rate as high as 40% (K4) (Supplementary Table 3). In terms of attitudes, the proportion of positive responses (“Strongly agree” and “Agree”) varied from 2.42 to 82.33%. The highest level of agreement (82.33%) was noted for the statement indicating that, although endometriosis is challenging to prevent, regular check-ups and an active lifestyle may mitigate the associated risk (A7). A noteworthy 97.58% of participants indicated concerns about a diagnosis of endometriosis and the potential necessity for surgical interventions (N while 97.1% reported experiencing anxiety upon receiving their diagnosis (A8) (Supplementary Table 4). Regarding practices, adherence to practice items, categorized as “Always” and “Often,” ranged from 37.05 to 82.08%. The highest level of adherence (82.08%) was recorded for following prescribed medication treatments as directed by a healthcare professional (P7), whereas the lowest adherence (37.05%) was observed for active participation in educational programs about endometriosis and postoperative care organized by medical institutions (P6). Concerning postoperative discomfort, 59.32% of participants opted to visit a hospital for treatment, 32.20% consulted a physician, and 5.08% chose to self-administer painkillers or anti-inflammatory medications (P8) (Supplementary Table 5).

### Univariate and multivariate logistic regression analysis

Multivariate logistic regression analysis indicated that, after controlling for age and other confounding variables, each 1-point increase in the knowledge score was associated with an 11% increase in the odds of demonstrating improved practice (OR = 1.11, 95% CI: 1.05–1.17, *P* < 0.001). In contrast, after adjusting for knowledge scores and additional covariates, each additional year of age was associated with a 3% decrease in the odds of exhibiting improved practice (OR = 0.97, 95% CI: 0.95–0.99, *P* = 0.008) (Table 2).

### Spearman correlation analysis

Spearman’s correlation analysis revealed significant positive relationships between knowledge and attitude (*r* = 0.105, *P* = 0.033), knowledge and practice (*r* = 0.175, *P* < 0.001), and attitude and practice (*r* = 0.100, *P* = 0.041) (Table 3).

**Table 3:** Spearman correlation analysis of KAP scores

	Knowledge	Attitude	Practice
Knowledge	1		
Attitude	0.105 (*P* = 0.033)	1	
Practice	0.175 (*P* < 0.001)	0.100 (*P* = 0.041)	1

### Structural equation model analysis

**Fig. 2:**
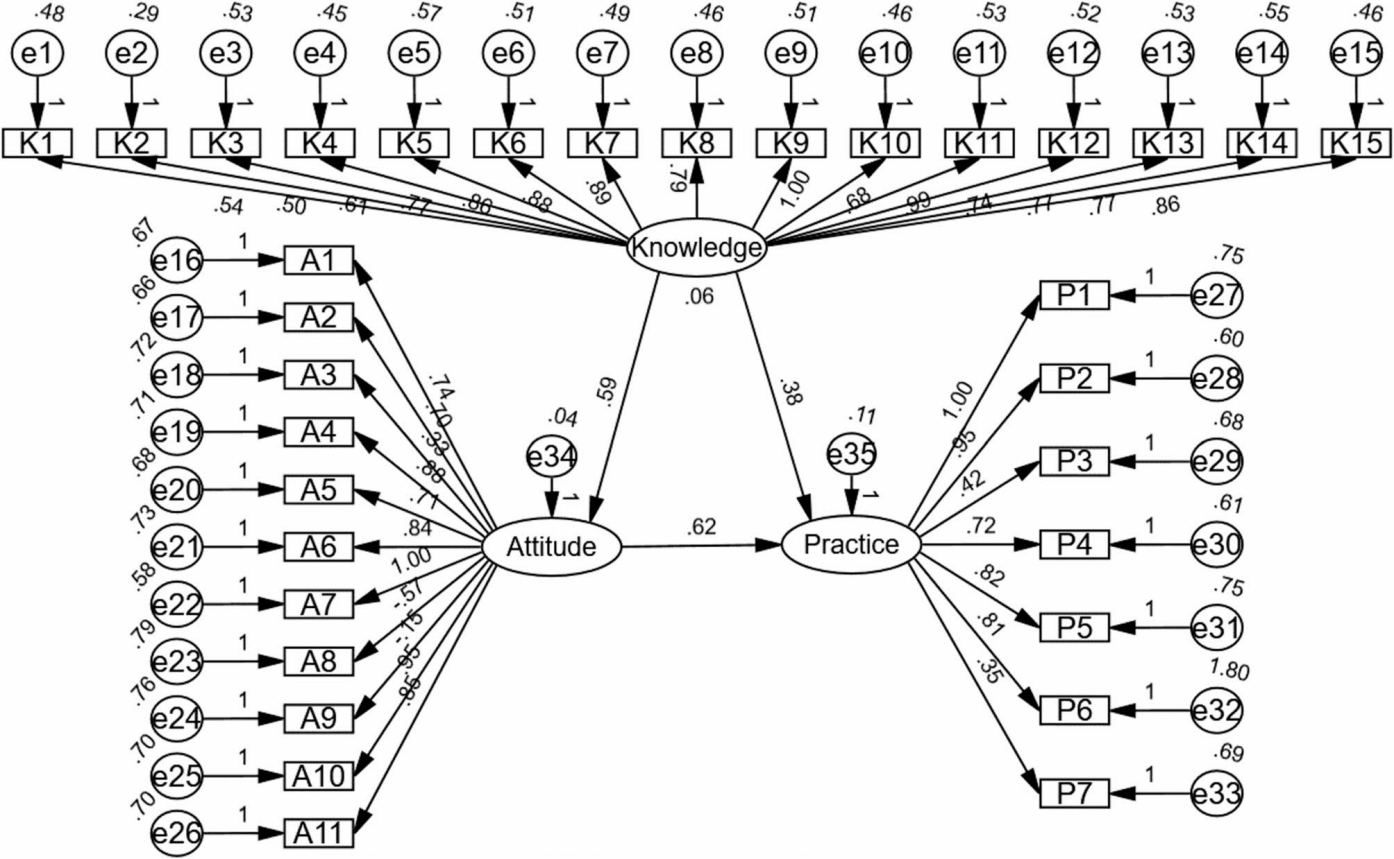
SEM model analysis results of KAP scores. All variables are observed variables. Direction of causality is indicated by single-headed arrows, and double-headed arrow indicates a correlation among variables. The standardized path coefficients are presented alongside the arrows

The SEM demonstrated highly favorable model fit indices: CMIN/DF value of 1.064 (Reference: 1–3 excellent, 3–5 good), RMSEA value of 0.012 (reference value < 0.08), IFI value of 0.911 (Reference > 0.08 good), TLI value of 0.894 (Reference: > 0.8 good), and CFI value of 0.901 (Reference: > 0.8 good), suggesting a well-fitting model (Table 4). The SEM indicated a significant direct path from knowledge to attitude, suggesting a potential influence, though causal interpretation is limited by the study’s cross-sectional design (β = 0.587, *P* = 0.003) (Table 5; Fig. 2).

**Table 4:** Model fit indices of structural equation model

Indicators	Reference	Actual
CMIN/DF	1–3: Excellent, 3–5: Good	1.064
RMSEA	< 0.08: Good	0.012
IFI	> 0.8: Good	0.911
TLI	> 0.8: Good	0.894
CFI	> 0.8: Good	0.901

*Abbreviations*: *CMIN/DF* Minimum Discrepancy of Confirmatory Factor Analysis/Degrees of Freedom, *RMSEA* Root Mean Square Error of Approximation, *IFI* Incremental Fit Index, *TLI* Tucker-Lewis Index, *CFI* Comparative Fit Index

**Table 5:** SEM results of KAP scores

Model path	β	*P*
Knowledge → Attitude	0.587	0.003
Attitude → Practice	0.615	0.061
Knowledge → Practice	0.378	0.161

## Discussion

### Knowledge gaps

Our study revealed that patients with endometriosis in Liaoning exhibited limited knowledge; however, they demonstrated positive attitudes and practices concerning surgical intervention and postoperative care. We observed significant positive correlations among the KAP scores, indicating that knowledge directly influences attitudes. Notable factors affecting KAP scores included smoking status, educational attainment, the presence of female relatives with endometriosis, and age.

The knowledge gaps observed among patients align with findings from other studies. For instance, research conducted in Pakistan indicated that women had limited knowledge regarding endometriosis, achieving a mean knowledge score of only 4.2 out of 10 prior to an educational intervention [30]. Despite these deficiencies, our participants demonstrated positive attitudes and practices concerning surgery and postoperative care. According to the theory of planned behavior, even in the presence of knowledge gaps, strong social and cultural expectations surrounding fertility can promote positive attitudes and compliance with medical recommendations [31]. In China, the cultural significance attributed to childbearing may drive patients to pursue surgical interventions and adhere to postoperative guidelines, notwithstanding their lack of knowledge. Similarly, a study in Denmark revealed that even healthcare professionals, such as nurses, maintained positive attitudes toward endometriosis care, even amidst knowledge deficits [24], highlighting that attitude can remain favorable despite limited disease knowledge— a pattern also observed among patients in our study. Furthermore, research in Poland showed that while women with endometriosis rated their overall quality of life higher than their perceived health, the physical domain received the lowest scores [32]. This suggests that, despite positive attitudes, a lack of understanding regarding the disease may result in a diminished quality of life. Our findings highlight the necessity for targeted educational initiatives to empower patients and improve health outcomes.

Most participants recognized the importance of seeking medical attention for progressively worsening symptoms, consistent with previous research indicating that women with endometriosis are more inclined to seek help for pelvic and menstrual pain [33]. However, there remains a substantial need for education regarding more subtle or atypical presentations of endometriosis. The alarming deficiency in awareness regarding the exacerbation of secondary dysmenorrhea as a primary symptom—acknowledged by merely 3.63% of participants—represents a significant finding, given that this symptom is critical for the prompt diagnosis and management of the condition [34]. This lack of awareness may arise from the overlap of endometriosis symptoms with other prevalent gynecological issues such as fibroids or pelvic inflammatory disease [35]. Therefore, enhancing education on the primary clinical signs of endometriosis is essential for improving early identification. The insufficient recognition of endometriosis as a chronic disease linked to elevated infertility rates—recognized by merely 12.11% of patients—highlights a substantial deficiency in educational efforts. Given that 30–50% of women with endometriosis experience infertility, this lack of awareness may contribute to delayed diagnoses and missed opportunities for fertility preservation [36]. To address this gap, it is vital to incorporate fertility counseling into routine clinical care, enabling patients to make informed reproductive decisions and consider early interventions to improve long-term fertility outcomes [37].

### Psychological impact

Most participants supported on regular check-ups and an active lifestyle as effective strategies to reduce the risk of endometriosis recurrence. This suggests that patients are cognizant of non-pharmacological methods to manage the condition, which aligns with the growing emphasis on integrated care models that combine medical treatments with lifestyle interventions [38]. Given the significance of family support among Chinese patients, incorporating family counseling could enhance adherence to recommended lifestyle changes and regular monitoring [39]. Conversely, some participants reported experiencing fear and anxiety upon receiving a diagnosis of endometriosis and the potential necessity for surgical treatment. According to the health belief model, such anxiety can stem from perceived threats, particularly concerns regarding infertility, surgical complications, and uncertainty about long-term outcomes [40]. The fear of compromised reproductive ability may exacerbate preoperative anxiety and influence decision-making regarding surgery [41]. Furthermore, the possibility of recurrence, which is common in endometriosis, can lead to feelings of helplessness and anxiety about the future [42]. These findings underscore the critical need for psychological support throughout the diagnosis and treatment process for endometriosis. Providing clear and empathetic explanations about procedures, their benefits and risks, expected outcomes, and fertility preservation options could alleviate patients’ fears and uncertainties.

In terms of practice, a high adherence rate to prescribed medication treatments was observed. Medication adherence is crucial for managing the chronic nature of endometriosis, particularly in alleviating symptoms such as pain, dysmenorrhea, and the risk of infertility [43]. However, a limited number of patients participated in educational programs focused on endometriosis and postoperative care. Several factors may elucidate this low participation rate. First, a lack of awareness regarding available educational programs may impede patients from seeking involvement. Second, time constraints, geographic accessibility, and the burden of ongoing medical appointments may further dissuade participation. Healthcare providers should actively promote educational programs through various channels, including social media and patient support groups [44]. Interactive workshops, case-based discussions, and peer-led support groups are recommended to enhance participation [45]. When confronted with postoperative discomfort, the majority expressed a strong reliance on professional medical care. Prompt consultation with healthcare providers can effectively address these issues, reduce the risk of long-term complications, and ensure proper healing [46]. Notably, a small proportion opted for self-medication with painkillers or anti-inflammatory medications. Given the delicate nature of post-surgical healing, improper self-medication could obscure symptoms of infection or internal bleeding. Follow-up appointments must be emphasized as a critical component of the recovery process, with clear instructions on which symptoms to report and how to manage discomfort at home.

### Factors affecting KAP

The robust positive correlations observed among the dimensions of KAP align with the foundational principles of the KAP Model [23, 47]. The results of our SEM analysis elucidated this relationship by demonstrating that knowledge exerts a direct and positive influence on attitudes (β = 0.587, *p* = 0.003). This finding underscores the theoretical justification for prioritizing educational interventions aimed at enhancing knowledge. This finding illustrates a synergistic chain reaction: enhanced knowledge improves threat perception and self-efficacy, which subsequently cultivates attitudes conducive to health-promoting behaviors. Specifically, patients with endometriosis who comprehended the risks, benefits, and recovery protocols associated with surgical interventions developed evidence-based positive attitudes, such as perceived severity and self-efficacy. These attitudes served as motivators for proactive practices, including adherence to hormonal therapy and pelvic floor exercises, with the perceived benefits outweighing the barriers. Structural equation modeling further validated a direct pathway from knowledge to attitude, emphasizing the critical role of education in shaping psychological responses. Several key factors influenced KAP: (1) Smokers demonstrated lower knowledge scores, likely attributable to barriers in healthcare access [48]; (2) Higher levels of education were predictive of more positive attitudes, presumably due to increased health literacy [49]; (3) Patients without a family history of endometriosis exhibited less favorable attitudes, indicating potential gaps in awareness; and (4) Older age was associated with decreased treatment adherence, potentially due to physical and cognitive limitations [50]. Notably, demographic variables did not reveal significant associations with attitudes, which may be ascribed to unmeasured psychosocial or cultural factors within our regression model.

This study has several limitations. The cross-sectional design restricts causal inferences between KAP and demographic factors. Longitudinal studies are needed to assess the long-term effects of KAP interventions on endometriosis patients. Additionally, the sample was drawn from a single region, which may not be representative of other regions or healthcare settings. Self-reported data may introduce social desirability bias, potentially inflating KAP scores [51].

## Conclusions

In conclusion, this study elucidates a significant disconnect among endometriosis patients in Liaoning Province. Despite exhibiting positive attitudes and practices toward treatment, there exists a notable deficiency in their fundamental understanding of the disease and its management. The positive correlations identified between knowledge, attitudes, and practices—particularly the direct influence of knowledge on attitudes—underscore a clear opportunity for intervention. Therefore, we advocate for the development and implementation of targeted educational programs. These initiatives should not only focus on disseminating information but also aim to empower patients, align their expectations with clinical realities, and ultimately enhance long-term adherence and health outcomes following surgical intervention for endometriosis.

## Supplementary Information

Supplementary Material 1.

Supplementary Material 2.

## Data Availability

All data generated or analysed during this study are included in this published article.

## Abbreviations

KAP: Knowledge, attitudes, and practices
DIE: Deep infiltrating lesions
SD: Standard deviations
IQR: Interquartile ranges
SEM: Structural equation modeling
