# Supplementary material for: Knowledge, attitudes, and practices of patients with endometriosis regarding endometriosis surgery and postoperative care in Liaoning Province, China: a cross-sectional analysis
Source: BMC Pregnancy Childbirth. 2025 Jul 28;25:795. doi: 10.1186/s12884-025-07852-1 (PMC12306003; doi:10.1186/s12884-025-07852-1)
Supplement: Supplementary file 1 — Supplementary Material 1. [file 12884_2025_7852_MOESM1_ESM.docx]

| Questionnaire ID |
| --- |
| Dear Participant,  We are researchers from XXX and sincerely invite you to participate in our study. This research aims to understand patients' knowledge, attitudes, and practices regarding endometriosis surgery and postoperative care, providing a basis for developing scientific intervention strategies. This may help more people in the future and improve their health conditions. Your participation in this study is voluntary. The study has been reviewed and approved by the Ethics Committee. If you agree to participate, please refer to the following instructions.  1.Please complete the questionnaire. There are no right or wrong answers—you only need to answer based on your actual situation. If you have any questions during the process, feel free to ask us. Once completed, please submit it promptly.  2.This study is a simple questionnaire survey and will not cause any harm to your physical or psychological well-being. However, it involves some personal information, such as your gender and age. We will strictly maintain confidentiality and ensure that your information is not disclosed, so please feel at ease while filling it out.  3.As a participant, you have the right to access information related to this study and its progress at any time. If you decide to withdraw from the study, please inform us, and your data will not be included in the research results.  Finally, we sincerely appreciate your valuable time and support for our scientific research!  □I acknowledge and agree that the collected data will be used for scientific research.  Informed Consent Signature:  Date of Participation: ____ Year ____ Month ____ Day |

| **Part 1 Basic Information** | |
| --- | --- |
| **1.Your age: ____ years old.** | |
| **2.Your educational level:** | a. Primary school or below b. Junior high school c. Senior high school/Technical secondary school d. Associate/Bachelor’s degree e. Master’s degree or above |
| **3.Your ethnicity:** | a. Han b. Ethnic minority |
| **4.Your employment status:** | a. Employed b. Unemployed c. Retired d. Self-employed e. Other |
| **5.Your average monthly per capita household income in the past year (including in-kind income and rental income):** | a.<2000  b.2000-5000  c.5000-10000  d.10000-20000  e.>20000  f. Prefer not to disclose |
| **6.Your marital status:** | a. Single b. Married c. Divorced d. Widowed |
| **7.Do you have children?** | a. Yes b. No |
| **8.Do you smoke?** | a. Never smoked b. Used to smoke c. Currently smoke |
| **9.Do you drink alcohol?** | a. Never drink b. Used to drink c. Currently drink |
| **10.Do you have medical insurance or other commercial insurance?** | a. Yes b. No |
| **11.How long has it been since your diagnosis of endometriosis?** | a. <1 year b. 1–3 years c. 3–5 years d. >5 years |
| **12.Do you have female relatives with endometriosis?** | a. Yes b. No c. Not sure |
| **13.Have you undergone surgical treatment for endometriosis?** | a. Yes b. No |

| **Part 2 Knowledge of Endometriosis Surgery and Postoperative Care** | | | |
| --- | --- | --- | --- |
| 1.Endometriosis (EMT) refers to the growth of endometrial tissue (glands and stroma) outside the uterine cavity and myometrium. | a. Very familiar | b. Heard of it | c. Not clear |
| 2.The primary symptom of endometriosis is progressively worsening secondary dysmenorrhea, commonly affecting women of reproductive age, between 25 and 45 years old. | a. Very familiar | b. Heard of it | c. Not clear |
| 3.Some patients with endometriosis do not exhibit any symptoms. | a. Very familiar | b. Heard of it | c. Not clear |
| 4.Endometriosis is a chronic disease with an infertility rate as high as 40%. | a. Very familiar | b. Heard of it | c. Not clear |
| 5.If you experience progressively worsening dysmenorrhea, abnormal menstruation, severe abdominal pain, dyspareunia, or difficulty conceiving, you should seek medical attention as soon as possible. | a. Very familiar | b. Heard of it | c. Not clear |
| 6.Indications for endometriosis surgery include ineffective drug treatment, worsening local lesions or persistent infertility, and ovarian endometriotic cysts with a diameter of ≥4 cm. | a. Very familiar | b. Heard of it | c. Not clear |
| 7.Laparoscopy is currently recognized as the best diagnostic method for endometriosis. | a. Very familiar | b. Heard of it | c. Not clear |
| 8.Endometriosis surgery is classified into conservative, semi-radical, and radical procedures. | a. Very familiar | b. Heard of it | c. Not clear |
| 9.The optimal treatment for endometriosis is laparoscopic surgery combined with medication. | a. Very familiar | b. Heard of it | c. Not clear |
| 10.Except for radical surgery, endometriosis has a high recurrence rate. | a. Very familiar | b. Heard of it | c. Not clear |
| 11.Endometriosis patients should monitor their symptoms and disease progression in daily life. Long-term medication users should also be aware of potential adverse drug reactions. | a. Very familiar | b. Heard of it | c. Not clear |
| 12.The prevention of endometriosis is limited, but timely treatment of reproductive tract disorders, oral contraceptive use, and regular exercise may help reduce the risk. | a. Very familiar | b. Heard of it | c. Not clear |
| 13.To relieve pain, patients should rest in bed as much as possible during menstruation. In severe cases, anti-inflammatory and analgesic medications may be taken orally or administered rectally. | a. Very familiar | b. Heard of it | c. Not clear |
| 14.Patients should engage in regular physical exercise and maintain a proper diet in daily life. | a. Very familiar | b. Heard of it | c. Not clear |
| 15.Patients should follow up as prescribed by their doctor, typically every 3 to 6 months. | a. Very familiar | b. Heard of it | c. Not clear |

| **Part 3 Attitudes Toward Endometriosis Surgery and Postoperative Care** | | | | | |
| --- | --- | --- | --- | --- | --- |
| 1.If a doctor recommends surgery for endometriosis, how willing are you to accept it?（P） | a. Strongly agree | b. Agree | c. Neutral | d. Disagree | e. Strongly disagree |
| 2.Do you trust the effectiveness and safety of surgical techniques for treating endometriosis?（P） | a. Strongly agree | b. Agree | c. Neutral | d. Disagree | e. Strongly disagree |
| 3.Do you believe that proper wound cleaning and postoperative care are crucial for preventing infections? (P) | a. Strongly agree | b. Agree | c. Neutral | d. Disagree | e. Strongly disagree |
| 4.Are you mentally prepared for a long-term battle with the disease?（P） | a. Strongly agree | b. Agree | c. Neutral | d. Disagree | e. Strongly disagree |
| 5.Do you believe that having sufficient knowledge about the disease helps with postoperative management of endometriosis? （P） | a. Strongly agree | b. Agree | c. Neutral | d. Disagree | e. Strongly disagree |
| 6.Do you think that timely surgical treatment and active daily postoperative management can help overcome the disease?（P） | a. Strongly agree | b. Agree | c. Neutral | d. Disagree | e. Strongly disagree |
| 7.Do you believe that although this disease is difficult to prevent, regular check-ups and an active lifestyle can reduce the risk?（P） | a. Strongly agree | b. Agree | c. Neutral | d. Disagree | e. Strongly disagree |
| 8.Did you feel anxious upon being diagnosed with endometriosis?（N） | a. Strongly agree | b. Agree | c. Neutral | d. Disagree | e. Strongly disagree |
| 9.Do you feel fear when facing the disease and potential surgical treatment? （N） | a. Strongly agree | b. Agree | c. Neutral | d. Disagree | e. Strongly disagree |
| 10.Do you believe that actively seeking psychological support can help manage the stress associated with surgery and postoperative care?（P） | a. Strongly agree | b. Agree | c. Neutral | d. Disagree | e. Strongly disagree |
| 11.Do you think that family understanding and encouragement are crucial for boosting confidence in overcoming the disease and reducing anxiety?（P） | a. Strongly agree | b. Agree | c. Neutral | d. Disagree | e. Strongly disagree |

| **Part 4 Behavioral Practices Related to Endometriosis Surgery and Postoperative Care** | | | | | |
| --- | --- | --- | --- | --- | --- |
| 1.Do you actively learn about endometriosis and its postoperative care?（P） | a. Always | b. Often | c. Occasionally | d. Rarely | e. Never |
| 2.Do you maintain a healthy diet, eating fresh vegetables, fruits, salmon, and walnuts while limiting high-fat meats? （P） | a. Always | b. Often | c. Occasionally | d. Rarely | e. Never |
| 3.Do you make an effort to regulate your emotions and positively cope with the physical and emotional challenges of endometriosis?（P） | a. Always | b. Often | c. Occasionally | d. Rarely | e. Never |
| 4.Do you undergo regular follow-up check-ups?（P） | a. Always | b. Often | c. Occasionally | d. Rarely | e. Never |
| 5.Do you engage in regular physical exercise?（P） | a. Always | b. Often | c. Occasionally | d. Rarely | e. Never |
| 6.Do you actively participate in educational programs on endometriosis and postoperative care organized by medical institutions?（P） | a. Always | b. Often | c. Occasionally | d. Rarely | e. Never |
| 7.Do you follow prescribed medication treatments as instructed by your doctor? （P） | a. Always | b. Often | c. Occasionally | d. Rarely | e. Never |
| 8.When experiencing postoperative discomfort, what is your first course of action?  a. Take painkillers or anti-inflammatory medication on your own b. Consult a doctor c. Visit a hospital for treatment d. Wait for symptoms to resolve on their own e. Seek advice from patients with similar experiences |  |  |  |  |  |

| **Thank you again for filling out our questionnaire, the information you provided will be valuable to us in the future!**  **Thank you for filling out our questionnaire！**  If you have any comments and suggestions on this survey, we would be honored to hear your voice.  Opinions and Suggestions： （optional）  In order to this questionnaire research can actually play a role in promoting the smooth development of the future return visit, if you are willing to leave your contact information, we would be grateful!  Your phone number： （optional） |
| --- |
