# Supplementary material for: Knowledge, attitudes, and practices of patients with endometriosis regarding endometriosis surgery and postoperative care in Liaoning Province, China: a cross-sectional analysis
Source: BMC Pregnancy Childbirth. 2025 Jul 28;25:795. doi: 10.1186/s12884-025-07852-1 (PMC12306003; doi:10.1186/s12884-025-07852-1)
Supplement: Supplementary file 2 — Supplementary Material 2. [file 12884_2025_7852_MOESM2_ESM.docx]

**Supplementary Table 1. Confirmatory factor analysis fitting indicators.**

| Indicators | Reference | Actual |
| --- | --- | --- |
| CMIN/DF | 1-3: Excellent, 3-5: Good | 1.064 |
| RMSEA | <0.08: Good | 0.012 |
| IFI | >0.8: Good | 0.911 |
| TLI | >0.8: Good | 0.894 |
| CFI | >0.8: Good | 0.901 |

**Supplementary Table 2. Confirmatory factor analysis coefficient.**

|  |  |  | **Estimate** | **Standardized Estimate** | **S.E.** | **C.R.** | **P** |
| --- | --- | --- | --- | --- | --- | --- | --- |
| K1 | <--- | Knowledge | 1.000 | 0.189 |  |  |  |
| K2 | <--- | Knowledge | 0.935 | 0.227 | 0.413 | 2.264 | 0.024 |
| K3 | <--- | Knowledge | 1.141 | 0.205 | 0.526 | 2.168 | 0.030 |
| K4 | <--- | Knowledge | 1.423 | 0.272 | 0.587 | 2.424 | 0.015 |
| K5 | <--- | Knowledge | 1.604 | 0.274 | 0.660 | 2.428 | 0.015 |
| K6 | <--- | Knowledge | 1.633 | 0.292 | 0.659 | 2.478 | 0.013 |
| K7 | <--- | Knowledge | 1.658 | 0.302 | 0.663 | 2.503 | 0.012 |
| K8 | <--- | Knowledge | 1.471 | 0.278 | 0.603 | 2.441 | 0.015 |
| K9 | <--- | Knowledge | 1.856 | 0.327 | 0.726 | 2.556 | 0.011 |
| K10 | <--- | Knowledge | 1.270 | 0.243 | 0.545 | 2.329 | 0.020 |
| K11 | <--- | Knowledge | 1.843 | 0.321 | 0.724 | 2.544 | 0.011 |
| K12 | <--- | Knowledge | 1.378 | 0.247 | 0.589 | 2.342 | 0.019 |
| K13 | <--- | Knowledge | 1.426 | 0.252 | 0.604 | 2.360 | 0.018 |
| K14 | <--- | Knowledge | 1.426 | 0.249 | 0.607 | 2.350 | 0.019 |
| K15 | <--- | Knowledge | 1.603 | 0.300 | 0.642 | 2.497 | 0.013 |
| A1 | <--- | Attitude | 1.000 | 0.224 |  |  |  |
| A2 | <--- | Attitude | 0.951 | 0.215 | 0.441 | 2.157 | 0.031 |
| A3 | <--- | Attitude | 0.444 | 0.097 | 0.355 | 1.249 | 0.212 |
| A4 | <--- | Attitude | 1.189 | 0.257 | 0.508 | 2.342 | 0.019 |
| A5 | <--- | Attitude | 0.962 | 0.213 | 0.448 | 2.147 | 0.032 |
| A6 | <--- | Attitude | 1.145 | 0.244 | 0.499 | 2.293 | 0.022 |
| A7 | <--- | Attitude | 1.358 | 0.318 | 0.537 | 2.530 | 0.011 |
| A8 | <--- | Attitude | -0.768 | -0.159 | 0.424 | -1.812 | 0.070 |
| A9 | <--- | Attitude | -0.209 | -0.045 | 0.338 | -0.617 | 0.538 |
| A10 | <--- | Attitude | 1.293 | 0.279 | 0.534 | 2.419 | 0.016 |
| A11 | <--- | Attitude | 1.148 | 0.249 | 0.497 | 2.310 | 0.021 |
| P1 | <--- | Practice | 1.000 | 0.423 |  |  |  |
| P2 | <--- | Practice | 0.954 | 0.446 | 0.223 | 4.283 | <0.001 |
| P3 | <--- | Practice | 0.419 | 0.202 | 0.158 | 2.657 | 0.008 |
| P4 | <--- | Practice | 0.724 | 0.352 | 0.187 | 3.878 | <0.001 |
| P5 | <--- | Practice | 0.819 | 0.358 | 0.209 | 3.911 | <0.001 |
| P6 | <--- | Practice | 0.811 | 0.238 | 0.269 | 3.012 | 0.003 |
| P7 | <--- | Practice | 0.355 | 0.170 | 0.154 | 2.305 | 0.021 |

**Supplementary Table 3. Knowledge dimension distribution.**

|  | **Very familiar n(%)** | **Heard of n (%)** | **Unclear n (%)** |
| --- | --- | --- | --- |
| **1. Endometriosis refers to the growth of endometrial tissue (glands and stroma) outside the uterine cavity and myometrium.** | 58 (14.04) | 166 (40.19) | 189 (45.76) |
| **2. The primary symptom of endometriosis is progressively worsening secondary dysmenorrhea, commonly affecting women of reproductive age, between 25 and 45 years old.** | 15 (3.63) | 234 (56.66) | 164 (39.71) |
| **3. Some patients with endometriosis do not exhibit any symptoms.** | 64 (15.5) | 120 (29.06) | 229 (55.45) |
| **4. Endometriosis is a chronic disease with an infertility rate as high as 40%.** | 50 (12.11) | 135 (32.69) | 228 (55.21) |
| **5. If you experience progressively worsening dysmenorrhea, abnormal menstruation, severe abdominal pain, dyspareunia, or difficulty conceiving, you should seek medical attention as soon as possible.** | 220 (53.27) | 113 (27.36) | 80 (19.37) |
| **6. Indications for endometriosis surgery include ineffective drug treatment, worsening local lesions or persistent infertility, and ovarian endometriotic cysts with a diameter of ≥4 cm.** | 87 (21.07) | 172 (41.65) | 154 (37.29) |
| **7. Laparoscopy is currently recognized as the best diagnostic method for endometriosis.** | 92 (22.28) | 187 (45.28) | 134 (32.45) |
| **8. Endometriosis surgery is classified into conservative, semi-radical, and radical procedures.** | 89 (21.55) | 205 (49.64) | 119 (28.81) |
| **9. The optimal treatment for endometriosis is laparoscopic surgery combined with medication.** | 130 (31.48) | 175 (42.37) | 108 (26.15) |
| **10. Except for radical surgery, endometriosis has a high recurrence rate.** | 89 (21.55) | 211 (51.09) | 113 (27.36) |
| **11. Endometriosis patients should monitor their symptoms and disease progression in daily life. Long-term medication users should also be aware of potential adverse drug reactions.** | 128 (30.99) | 170 (41.16) | 115 (27.85) |
| **12. The prevention of endometriosis is limited, but timely treatment of reproductive tract disorders, oral contraceptive use, and regular exercise may help reduce the risk.** | 81 (19.61) | 166 (40.19) | 166 (40.19) |
| **13. To relieve pain, patients should rest in bed as much as possible during menstruation. In severe cases, anti-inflammatory and analgesic medications may be taken orally or administered rectally.** | 104 (25.18) | 175 (42.37) | 134 (32.45) |
| **14. Patients should engage in regular physical exercise and maintain a proper diet in daily life.** | 185 (44.79) | 146 (35.35) | 82 (19.85) |
| **15. Patients should follow up as prescribed by their doctor, typically every 3 to 6 months.** | 165 (39.95) | 180 (43.58) | 68 (16.46) |

**Supplementary Table 4. Attitude dimension distribution.**

|  | **Strongly agree**  **n (%)** | **Agree**  **n (%)** | **Neutral**  **n (%)** | **Disagree**  **n (%)** | **Strongly disagree**  **n (%)** |
| --- | --- | --- | --- | --- | --- |
| **1. If a doctor recommends surgery for endometriosis, how willing are you to accept it?** | 178 (43.10) | 138 (33.41) | 91 (22.03) | 4 (0.97) | 2 (0.48) |
| **2. Do you trust the effectiveness and safety of surgical techniques for treating endometriosis?** | 186 (45.04) | 144 (34.87) | 76 (18.40) | 4 (0.97) | 3 (0.73) |
| **3. Do you believe that proper wound cleaning and postoperative care are crucial for preventing infections?** | 199 (48.18) | 124 (30.02) | 83 (20.10) | 4 (0.97) | 3 (0.73) |
| **4. Are you mentally prepared for a long-term battle with the disease?** | 201 (48.67) | 124 (30.02) | 79 (19.13) | 5 (1.21) | 4 (0.97) |
| **5. Do you believe that having sufficient knowledge about the disease helps with postoperative management of endometriosis?** | 211 (51.09) | 111 (26.88) | 86 (20.82) | 3 (0.73) | 2 (0.48) |
| **6. Do you think that timely surgical treatment and active daily postoperative management can help overcome the disease?** | 208 (50.36) | 122 (29.54) | 74 (17.92) | 3 (0.73) | 6 (1.45) |
| **7. Do you believe that although this disease is difficult to prevent, regular check-ups and an active lifestyle can reduce the risk?** | 205 (49.64) | 135 (32.69) | 68 (16.46) | 3 (0.73) | 2 (0.48) |
| **8. Did you feel anxious upon being diagnosed with endometriosis?** | 194 (46.97) | 122 (29.54) | 85 (20.58) | 7 (1.69) | 5 (1.21) |
| **9. Do you feel fear when facing the disease and potential surgical treatment?** | 212 (51.33) | 114 (27.60) | 77 (18.64) | 7 (1.69) | 3 (0.73) |
| **10. Do you believe that actively seeking psychological support can help manage the stress associated with surgery and postoperative care?** | 176 (42.62) | 144 (34.87) | 83 (20.10) | 5 (1.21) | 5 (1.21) |
| **11. Do you think that family understanding and encouragement are crucial for boosting confidence in overcoming the disease and reducing anxiety?** | 198 (47.94) | 121 (29.30) | 86 (20.82) | 5 (1.21) | 3 (0.73) |

**Supplementary Table 5. Practice dimension distribution.**

|  | **Always n (%)** | **Often n (%)** | **Occasionally n (%)** | **Rarely n (%)** | **Never n (%)** |
| --- | --- | --- | --- | --- | --- |
| **1. Do you actively learn about endometriosis and its postoperative care?** | 189 (45.76) | 121 (29.30) | 83 (20.10) | 13 (3.15) | 7 (1.69) |
| **2. Do you maintain a healthy diet, eating fresh vegetables, fruits, salmon, and walnuts while limiting high-fat meats?** | 181 (43.83) | 139 (33.66) | 83 (20.10) | 6 (1.45) | 4 (0.97) |
| **3. Do you make an effort to regulate your emotions and positively cope with the physical and emotional challenges of endometriosis?** | 179 (43.34) | 143 (34.62) | 84 (20.34) | 4 (0.97) | 3 (0.73) |
| **4. Do you undergo regular follow-up check-ups?** | 197 (47.70) | 134 (32.45) | 74 (17.92) | 6 (1.45) | 2 (0.48) |
| **5. Do you engage in regular physical exercise?** | 189 (45.76) | 125 (30.27) | 80 (19.37) | 15 (3.63) | 4 (0.97) |
| **6. Do you actively participate in educational programs on endometriosis and postoperative care organized by medical institutions?** | 79 (19.13) | 74 (17.92) | 92 (22.28) | 91 (22.03) | 77 (18.64) |
| **7. Do you follow prescribed medication treatments as instructed by your doctor?** | 221 (53.51) | 118 (28.57) | 63 (15.25) | 9 (2.18) | 2 (0.48) |
| **8. When experiencing postoperative discomfort, what is your first course of action?** | **Take painkiller or anti-inflammatory medication on your own n (%)** | **Consult a doctor n (%)** | **Visit a hospital for treatment n (%)** | **Wait for symptoms to resolve on their own n (%)** | **Seek advice from patients with similar experiences n (%)** |
|  | 21 (5.08) | 133 (32.20) | 245 (59.32) | 14 (3.39) | 0 |
